# Do students learn to be more conscientious at medical school?

**DOI:** 10.1186/1472-6920-12-54

**Published:** 2012-07-11

**Authors:** Andrew T Chaytor, Jacqueline Spence, Ann Armstrong, John C McLachlan

**Affiliations:** 1 School of Medicine and Health, Durham University, Queens Campus, Thornaby, Stockton-on-Tees, UK

## Abstract

**Background:**

Professionalism in medical students is not only difficult to define but difficult to teach and measure. As negative behaviour in medical students is associated with post-graduate disciplinary action it would be useful to have a model whereby unprofessional behaviour at the undergraduate level can easily be identified to permit appropriate intervention. We have previously developed a scalar measure of conscientiousness, the Conscientiousness Index (CI), which positively correlates to estimates of professional behaviour in undergraduate medical students. By comparing CI points awarded in year 1 and year 2 of study we were able to use the CI model to determine whether teaching and clinical exposure had any effect on students’ conscientiousness.

**Methods:**

CI points were collected by administrative staff from 3 successive cohorts of students in years 1 and 2 of study. Points were awarded to students for activities such as submission of immunisation status and criminal record checks, submission of summative assignments by a specified date and attendance at compulsory teaching sessions. CI points were then converted to a percentage of maximal possible scores (CI %) to permit direct comparison between years 1 and 2 of study.

**Results:**

CI % scores were generally high with each year of study for each cohort showing negatively skewed normal distributions with peaks > 89%. There was a high degree of correlation of CI % scores between year 1 and year 2 of study for each cohort alone and when cohort data was combined. When the change in CI % from year 1 to year 2 for all students was compared there was no significant difference in conscientiousness observed.

**Conclusions:**

We have provided evidence that use of a CI model in undergraduate medical students provides a reliable measure of conscientiousness that is easy to implement. Importantly this study shows that measurement of conscientiousness by the CI model in medical students does not change between years 1 and 2 study suggesting that it is a stable characteristic and not modified by teaching and clinical exposure.

## Background

In the UK, there are published guidelines on professionalism, such as *Good Medical Practice*[1] and *Medical Students: Professionalism and Fitness to Practice*[1,2] available to medical students and medical educators. Nevertheless, professionalism in a medical context remains hard to define with over 90 attributes having been described [3]. This makes it difficult to teach and also difficult to measure [4]. Despite this, medical students do appear to comprehend what professional behaviour they are expected to display and are able to describe this in examinations [5].

A key question is whether professionalism is a fixed characteristic or whether it can be promoted over time. The implications of these two possibilities are profound as it will explore whether the teaching of professionalism has any effect on the professional behaviour exhibited in medical students and doctors.

Negative behaviour in undergraduate medical students has been associated with postgraduate disciplinary action [6-8]. The development of an effective measure of such behaviour particularly if implemented in the early stages of medical training, may thus help to identify individuals more likely to display unprofessional behaviour when practicing as a qualified doctor. This would permit early intervention to support such individuals or could be used to prevent progression to the later stages of a medical programme. However, previous attempts at measuring professionalism in students have relied upon subjective observations which do not permit easy comparison between students [9].

We have previously explored the relationship between an objective measure of the stable personality trait of conscientiousness and the construct of professionalism in undergraduate medical students [10,11]. This work showed that conscientiousness scores were positively correlated with both staff and student estimates of professionalism and enabled us to devise a scalar measure of conscientiousness, the Conscientiousness Index (CI) [10], which avoids the pitfalls of attempting to measure professionalism qualitatively [12].

Interestingly, lack of conscientious behaviour has previously been shown to be associated with unprofessional behaviour in clinical practice [13].

Points awarded to students that form the CI reflect the expectation that conscientious students would be dutiful, self-disciplined, highly organised and thorough in their approach to tasks. Constructing a student CI score therefore involves administrative staff collecting Yes/No decisions based on, for example, whether a student has attended a compulsory teaching session or submitted an assignment on time [10]. This simple objective approach is therefore easy to implement and avoids making complex subjective decisions.

We have previously reported a slight increase in CI% in a small number of students between years 1 and 2 of study [11] which would suggest that conscientious behaviour improves following exposure to teaching. In this paper, we have therefore expanded this study to explore the stability of the CI as a quantitative measure of conscientious behaviour in 3 cohorts of students which thus may also have implications for the stability of professionalism in undergraduate medical students.

## Methods

Ethical approval for the study was granted by the ethics committee of the School of Medicine and Health, University of Durham. Conscientiousness Index points were gathered from Phase I (Years 1 and 2) undergraduate medicine students at a UK medical school. Data were collected from 3 consecutive cohorts of students beginning their studies in the academic years 2006–7, 2007–8 and 2008–9.

### Conscientiousness Index

Details of the Conscientiousness Index are previously described [10]. In brief, at the start of the academic year students were informed verbally and via the VLE (Virtual Learning Environment) that recording of Conscientiousness Index points was to occur but that such points would not be used in formative or summative assessments. Students were awarded Conscientiousness Index points from October to April which were then recorded by administrative staff. Points were awarded for: attendance at compulsory teaching sessions, submission of immunisation status and criminal record checks, submission of summative assignments by a specified date, participating in required administrative tasks such as allocation of hospital base units and completing online course evaluation and feedback. Students that did not fulfill an obligation and provided a satisfactory explanation were not penalised in terms of awarding CI points. Conscientiousness Index points could also be deducted for adverse events such as failure to appropriately respond to staff following repeated e-mails or letters.

### Data analysis

**Figure 1:**
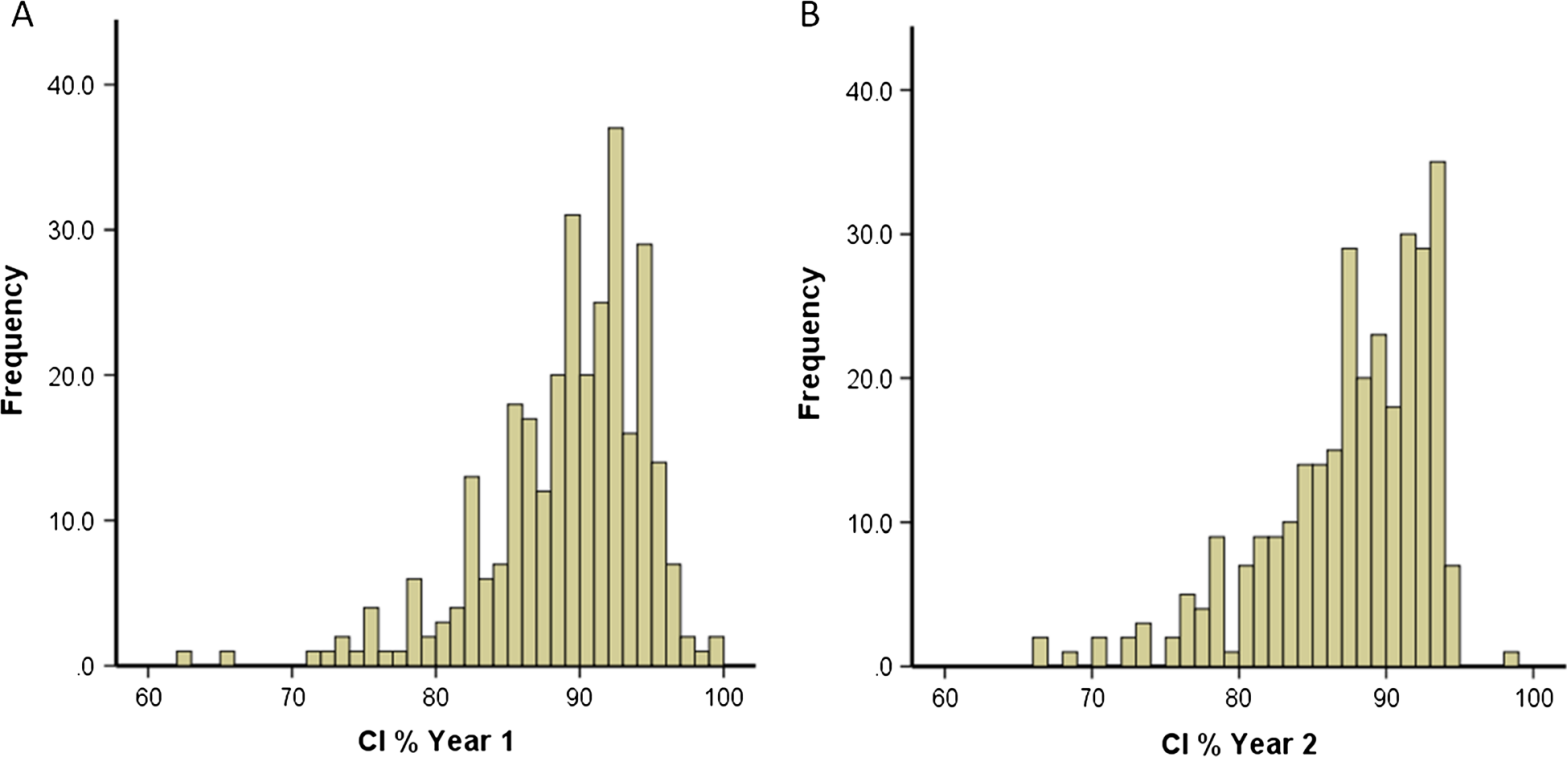
Distribution of CI points awarded to medical students in year 1 (A) and year 2 (B) of study.

**Figure 2:**
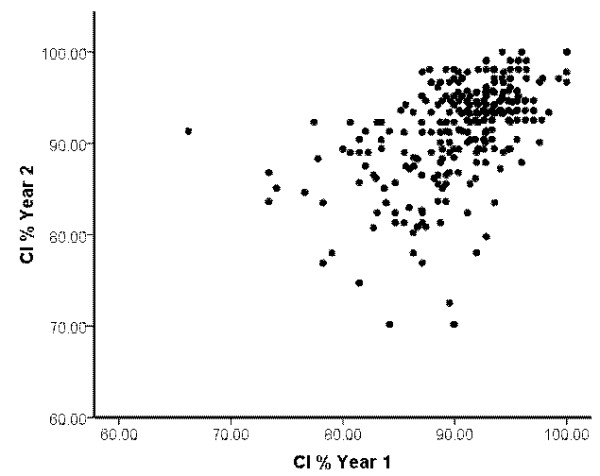
Scatter plot showing correlation between CI % scores in years 1 and 2 of study for the combined cohorts.

**Figure 3:**
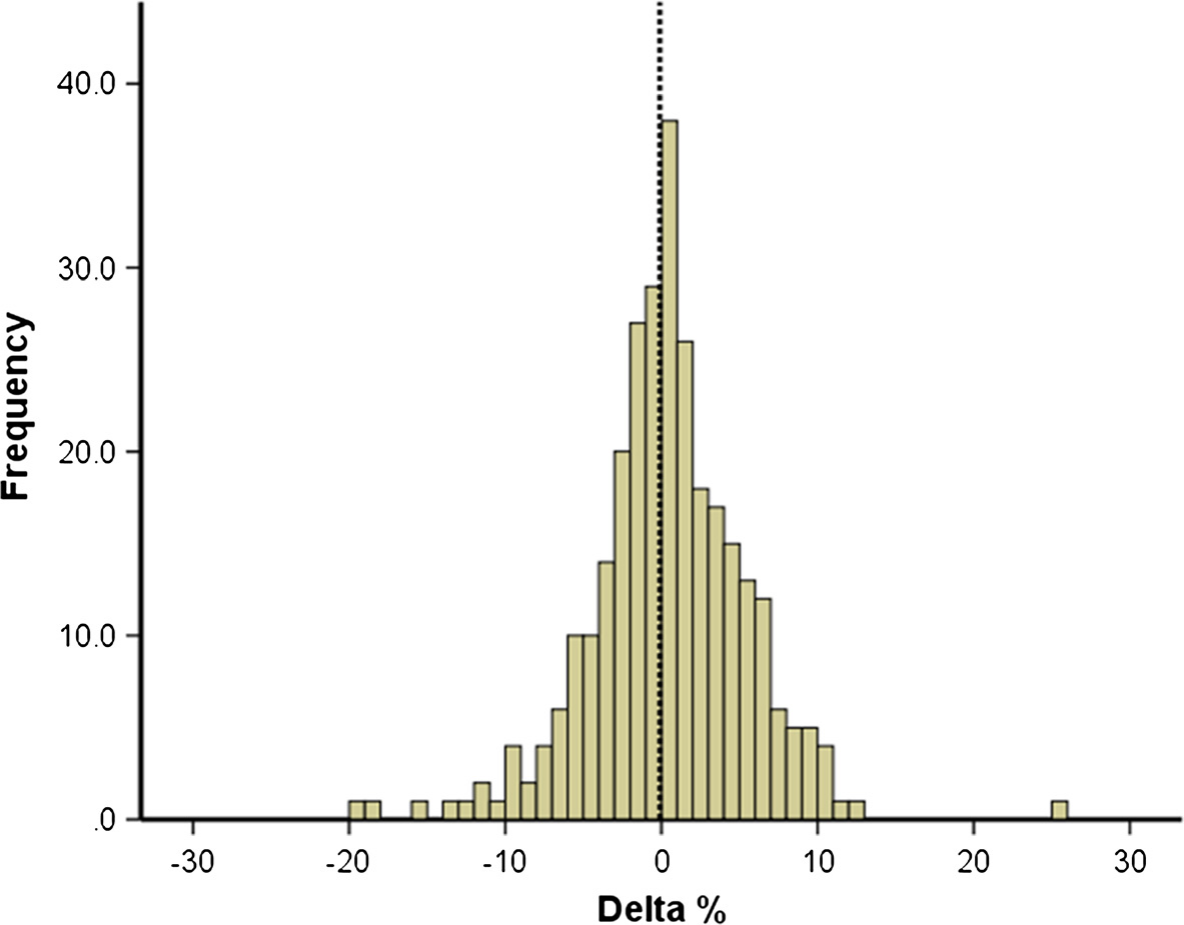
Change (delta %) in CI % scores from year 1 to year 2 of study for the combined cohorts.

The number of events in which Conscientiousness Index points could be awarded differed from cohort to cohort and from year 1 to year 2 within the same cohort of students. This was due to the number of compulsory sessions varying from one cohort to the next and also between years 1 and year 2 of study. Additionally, some events only occur at one particular time, for example, collection of immunisation status occurs in year 1 only. Points awarded were therefore expressed as a percentage of maximal possible points obtainable in a particular academic year to produce a Conscientiousness Index percentage score (CI %) thus permitting statistical comparison between years 1 and 2 of study. The change (delta) in CI % score from year 1 to year 2 was achieved by subtracting a student’s year 1 CI % from their year 2 CI % score. A positive delta value thus reflects an increase in Conscientiousness Index performance and a negative delta a decrease in performance. It was not possible to calculate the delta CI % for those students who did not progress from year 1 to year 2 or had deferred to year 2 from a previous year with no CI points recorded. In such cases the CI % scores have been retained in Figure 1 and Table 1 to give a more accurate reflection of CI % awarded in that year group, but were excluded from Figures 2 and 3 and Tables 2 and 3 where correlation between year 1 and year 2 scores or a change in CI % scores from year 1 to year 2 are shown.

**Table 1:** Descriptive statistics for CI points awarded to students in years 1 and 2 of study over 3 consecutive cohorts and when cohort data is combined

	**2006-7 cohort**				**2007-8 cohort**				**2008-9 cohort**				**Combined cohorts**			
	***n***	**Points range(max)**	**Mean %**	**S.D**	***n***	**Points range(max)**	**Mean %**	**S.D**	***n***	**Points range(max)**	**Mean %**	**S.D.**	***n***	**Points range(max)**	**Mean %**	**S.D.**
**Year 1**	110	87-134(140)	**89.8**	5.39	93	100-135(136)	**91.4**	4.8	102	91-124(128)	**86.2**	5.1	305	87-135(140)	**89.1**	5.54
**Year 2**	112	73-104(112)	**91.8**	5.59	91	76-94(95)	**91.2**	3.4	98	66-91(97)	**84.4**	5.9	301	66-104(112)	**89.2**	6.13

**Table 2:** Correlation of CI % awarded to students in years 1 and 2 of study over 3 consecutive cohorts and when cohort data is combined

	**2006-7 cohort**	**2007-8 cohort**	**2008-9 cohort**	**Combined cohorts**
***R* value**	0.494 ***	0.637***	0.530***	0.54***

*** Denotes a significant correlation with *P* <0.001.

**Table 3:** Change in CI % awarded to students from year 1 to year 2 of study for 3 consecutive cohorts and when cohort data is combined

	**2006-7 cohort**			**2007-8 cohort**			**2008-9 cohort**			**Combined cohorts**		
	***n***	**Mean delta**	**S.D.**	***n***	**Mean delta**	**S.D.**	***n***	**Mean delta**	**S.D.**	***n***	**Mean delta**	**S.D.**
**Change (delta) in CI% score**	107	**+1.84**	5.22	91	**−0.4**	3.5	98	**−1.56**	5.4	296	**−0.11**	5.1

Pearson’s correlation test was used to assess correlation between CI % scores for years 1 and 2 of study for each successive cohort. A one-sample Student’s *t*-test (mean set at zero) was used to assess the change between year 1 and year 2 CI % scores. SPSS Version 17.0 was used for statistical analysis of data with *P* < 0.05 considered statistically significant in all cases. Data is expressed as mean ± SD.

## Results

### Conscientiousness Index points

Conscientiousness index (CI) points were awarded in years 1 and 2 of study over 3 consecutive cohorts of undergraduate medical students beginning their studies in the academic years 2006–7, 2007–8 and 2008–9. The range of points obtained varied between year 1 and year 2 of study and between cohorts of students (Table 1). Conversion of CI points into a percentage of maximal possible scores (CI %) permitted comparison between years 1 and 2 of study within the same cohort of students and when data was combined from all 3 cohorts.

### Distribution of Conscientiousness Index % scores

Simple visual comparison of CI % scores for each of the 3 cohorts and for each year of study shows similar negatively skewed (towards the right) leptokurtic distributions with peaks at 93%, 95% and 89% for year 1 and 96–7%, 94% and 91% for year 2. Combining the CI % for each year of study showed similar distributions of scores with peaks at 93% and 94% for years 1 and 2 (Figure 1).

### Correlation between Conscientiousness Index % scores between years 1 and 2 of study

Figure 2 is a scatter plot of the combined data for all 3 cohorts of students. Analysis using a Pearson’s correlation test showed a high degree of correlation (*P* = 0.001, with *R* = 0.54; Table 2). There were similar significant correlations observed for each individual cohort of students (*P* = 0.001 for each cohort, with *R* = 0.494, 0.637 and 0.530 for the 2006–7, 2007–8 and 2008–9 cohorts, respectively; Table 2).

### Change (delta) in Conscientiousness Index % scores from year 1 to year 2

Figure 3 shows the distribution of the change (delta %) in CI % from year 1 to year 2 for the combined cohorts. The data is normally distributed around a value of zero which represents no change in CI % score. A positive delta % value reflects an increase in CI % and a negative value a reduction. There is no significant difference when examining the change in CI % from year 1 to year 2 for the combined cohorts (Figure 3; Table 3).

## Discussion

We have previously shown that the CI model correlates with teaching staff and peer estimates of professionalism in undergraduate students in a UK medical school [10,11] and have previously reported that in a single cohort of students there was a small increase in CI % [11] which could reflect an improvement in conscientious behaviour which may be attributable to teaching and clinical exposure. However, we have now expanded this study to include 3 consecutive cohorts thus considerably increasing the pool of students in which conscientiousness points were observed. There was some minor variation between cohorts but when the data is pooled there is no significant change in CI % scores between years 1 and 2 of studies. Our findings in this paper demonstrate that conscientious behaviour in medical students, as measured by using a conscientiousness index, does not significantly change from year 1 to year 2 of studies suggesting that the CI is measuring the stable trait of conscientiousness and is not modified by teaching.

The trait of conscientiousness is listed as one of the ‘Big Five’ domains of personality [14] and has been shown to positively correlate to exam performance in UK university students when measured qualitatively by personal inventory [15]. Similar approaches have found that conscientiousness significantly predicts final scores in examinations in pre-clinical medical students with those students who scored low in conscientiousness more likely to gain an unsuccessful outcome [16]. Conscientiousness is also a good predictor of job performance [17,18] and this may be of particular importance in the medical profession where as previously mentioned a lack of conscientious behaviour has been associated with unprofessional behaviour in clinical practice [13]. Other ‘Big Five’ personality traits may also have a role to play as predictors of professionalism such as agreeableness, as could some facets of traits such as warmth and positive emotions (extroversion) and ideas (openness to experience). However, the problem arises here of how to easily and reliably measure other traits and facets as this is normally attempted by qualitative methods by use of questionnaires. The collection of CI points producing a scalar measure of conscientiousness provides a situated measure of this behaviour in the context of the early stages of study in undergraduate medicine. It is possible that the CI may also be measuring a different aspect of this trait compared to the more generalised measures of the ‘Big Five’ personal inventory. However, even if the CI is measuring something ‘different’ the findings presented in this study, together with previously publish data, suggest the CI model may have an important role in identifying early on a lack of conscientious behaviour in medical students [10,11]. Further validation of the reliability of the CI may be achieved by adoption of similar approaches in other professions where identifying problematic behaviour at an early stage would be of benefit. Advantages of employing the CI model are its ease of collection, production of a quantitative outcome allowing direct comparison between students and the fact that it records actual student behaviour. Conscientiousness may not be the only personality trait that contributes towards professional behaviour but it is one that is perhaps the most important and easy to measure [10]. Although the CI may not directly measure professional behaviour it may play an important role in identifying individuals more likely to exhibit negative behaviour in the workplace.

CI % scores were generally high, with mean values of ~90% for both years 1 and 2 for the combined data from all three cohorts (Figures 1 and 2; Table 1) which suggests that medical students are on the whole a highly conscientious grouping. Overall, in addition to CI being reported to be stable within a single academic year [10] this study shows similar negatively skewed distributions for both years 1 and 2 of study (Figure 1). This suggests the CI % is stable and although there are different components contributing to the final CI points totals, a similar trait of conscientiousness is being measured in both years 1 and 2 for each cohort. This is further reinforced by the significant positive correlation of year 1 and year 2 CI % scores observed in Figure 2 and Table 2. The observed correlation of 0.54 for the combined cohorts (Table 2) would represent a significant effect size in educational terms [19]. The remaining variance may reflect the degree of change between individuals and cohorts, as in one cohort the mean CI % scores show a small increase and in the other two cohorts it decreases slightly with a net overall effect of virtually no change for the combined cohorts (Figure 3; Table 3). This provides further evidence for the reliability of the CI as a measure of conscientious behaviour and raises the interesting question of why the mean CI % scores did not indeed increase in year 2 overall.

It may be argued that an increase in mean CI % scores would be expected to occur in year 2, as previously reported [11], as students are exposed to further teaching, are further exposed to the concept of professional behaviour in doctors and their peers and experience more patient contact. However, as personality is thought to be stable during adulthood [20] conscientiousness may be difficult to modify in medical students without specific intervention. This could potentially raise the issue of preventing future problems of negative behaviour in practice by screening out likely individuals with low conscientiousness at early stages of the undergraduate curriculum or even at the admissions stage to medical school. Using conscientiousness as a screening mechanism during the admissions process would require a CI model to have been implemented at the students previous educational institution or workplace. This may be difficult to achieve in secondary education in the UK (normally up to age 18), with the reliability of the data also questionable as personality is reported as being stable post 18 years [20], but would be less problematic for graduate students where CI data could more easily be collected. It is also possible that applicants to medical schools could fake the desired personality traits at interview but use of a CI model would measure actual conscientiousness longitudinally and not just that displayed on a single occasion. This could be used as an argument for offering places at medical school only to graduate students with a satisfactory conscientiousness profile. However, we have previously reported that there was no difference in the CI % between medical students who were graduates and those that had commenced their medical studies directly after completing secondary education [11]. One further possibility why the CI did not significantly change from year 1 to year 2 of studies is that patient contact occurs early (week 2) in the first year of the medical programme at our institution so this may act as a switching-on for the requirement of professional behaviour from an early stage.

In the 2007–8 and 2008–9 cohorts virtually all students who performed in the top half of the class for CI in year 1 show a decrease in CI % score for year 2 (data not shown) with the opposite trend seen in the 2006–7 cohort. This effect may be due to regression to the mean [21] as the combined data shows no significant overall change in CI % scores (Table 3). As conscientiousness is also linked to exam performance [16] it is also possible that students with low a CI in year 1 also performed poorly in exams and put extra effort into year 2 studies, for example, in the form of increased attendance which would improve CI scores. The components comprising the possible total CI points available for year 2 also differs from year 1 so it may be more difficult to achieve the highest scores and a levelling out of CI points occurred. The high CI scores generally achieved by students may have been due to the relative ‘ease’ at which students could accumulate some points and may have contributed towards a ceiling effect on the data obtainable. However, the range of scores achieved (Table 2) and the number of different categories of data collected (up to 19 for year 1 and 12 for year 2) suggest this is more likely to reflect that medical students are a highly conscientious group and thus achieve high CI scores. Further analysis of the components of the CI index are required to further explore this, which lies out with the scope of this study.

Although we have reported no change in CI score and hence conscientious behaviour between year 1 and year 2 of studies it has been reported that in adulthood, up to the age of ~40 years, conscientiousness, aspects of extroversion (social dominance and emotional stability), open mindedness and agreeableness all improve [22,23]. This suggests that conscientiousness may indeed improve in the later years of undergraduate study and/or post-graduation. The relative components of personality that contribute towards professional behaviour may thus also vary with age. For example, does conscientiousness remain stable while other traits and facets change altering the relative contribution of conscientiousness towards professional behaviour. To provide further evidence of the validity and reliability of the CI as a measure of conscientiousness an extension of the project into the later stages of undergraduate medical training and beyond is therefore required. This would map out a students’ conscientiousness throughout their entire medical training programme. It may then be possible to determine more definitively the predictive validity of the CI in highlighting individuals at risk of experiencing postgraduate disciplinary procedures and importantly determine more precisely whether conscientiousness remains a stable characteristic throughout medical school unaffected by teaching. The use of a CI may also be of use in other professions such as teaching where it would be an advantage to flag negative behaviour in individuals at an early stage in training.

## Conclusions

This study shows that use of a CI model in undergraduate medical students provides a reliable measure of conscientiousness which may provide an indicator of unprofessional behaviour in students and importantly may identify at an early stage in their training those individuals who are more likely to exhibit unprofessional behaviour in future practice. This would allow early intervention and support for such individuals. Importantly our data shows that conscientiousness, as measured by the CI, in medical students did not change between years 1 and 2 of study suggesting that it is a stable characteristic and not modified by teaching and clinical exposure.

## Competing interests

The author(s) declare that they have no competing interests.

## Authors' contributions

JM conceived and designed study. AA and JS collected data. AC evaluated and analysed data and wrote first draft of paper. JM and AC contributed to final version of paper. All authors read and approved the final manuscript.

## Pre-publication history

The pre-publication history for this paper can be accessed here:

http://www.biomedcentral.com/1472-6920/12/54/prepub
